# Conditionally Site-Independent Neural Evolution of Antibody Sequences

**Published:** 2026-03-15

**Authors:** Stephen Zhewen Lu, Aakarsh Vermani, Kohei Sanno, Jiarui Lu, Frederick A. Matsen, Milind Jagota, Yun S. Song

**Affiliations:** 1 University of California, Berkeley; 2 Mila - Québec AI Institute; 3 Université de Montréal; 4 Fred Hutchinson Cancer Research Center; 5 University of Washington; 6 Howard Hughes Medical Institute; 7 Columbia University

## Abstract

Common deep learning approaches for antibody engineering focus on modeling the marginal distribution of sequences. By treating sequences as independent samples, however, these methods overlook affinity maturation as a rich and largely untapped source of information about the evolutionary process by which antibodies explore the underlying fitness landscape. In contrast, classical phylogenetic models explicitly represent evolutionary dynamics but lack the expressivity to capture complex epistatic interactions. We bridge this gap with **COSINE**, a continuous-time Markov chain parameterized by a deep neural network. Mathematically, we prove that COSINE provides a first-order approximation to the intractable sequential point mutation process, capturing epistatic effects with an error bound that is quadratic in branch length. Empirically, COSINE outperforms state-of-the-art language models in zero-shot variant effect prediction by explicitly disentangling selection from context-dependent somatic hypermutation. Finally, we introduce *Guided Gillespie*, a classifier-guided sampling scheme that steers COSINE at inference time, enabling efficient optimization of antibody binding affinity toward specific antigens.

## 1. Introduction

Antibodies are key effectors of the adaptive immune response, enabling humans and other vertebrates to recognize and neutralize an enormous diversity of molecular targets (antigens). This diversity is made possible by an accelerated evolutionary process operating within individuals, known as *affinity maturation*. During an immune response, B cells evolve in germinal centers, where their antibody genes undergo rapid somatic hypermutation (SHM) and are subsequently subjected to selection that favors variants with improved antigen binding. Repeated cycles of mutation and selection give rise to clonal trees of antibody variants, in which strongly binding lineages expand and diversify, while weak binders are progressively eliminated.

Affinity maturation thus represents a rare example of evolution that is both rapid and experimentally observable, making it an attractive system for studying how mutation and selection jointly shape protein function. To observe the results of this process, many studies have leveraged high-throughput sequencing to generate large-scale profiling of antibody repertoires from peripheral blood, yielding hundreds of thousands of antibody sequences per individual across diverse immune contexts. These data, rich in mature antibody sequences, have been used to train a plethora of antibody sequence models (Graves et al., 2020; Ruffolo et al., 2021; Leem et al., 2021; Olsen et al., 2022b; Hie et al., 2023; Kenlay et al., 2024b; Olsen et al., 2024a; Shanker et al., 2024; Wang et al., 2025). While such models have demonstrated impressive performance in capturing epistatic constraints within antibody sequences, they fundamentally lack the ability to model the time-dependent *process* over which antibodies mature. In particular, general antibody language models are trained under the assumption that the observed sequences in the clonal tree are *independent and identically distributed (i.i.d.)* samples from a stationary distribution. In reality, however, antibody sequences emerge through a structured evolutionary process as mutated descendants of specific germline progenitors. As a result, the strong performance of these models may in part stem from memorization of conserved germline residues rather than from a true understanding of the complex *process* of affinity maturation itself (Ng & Briney, 2024; Olsen et al., 2024a).

Developing principled and expressive models of sequence evolution that capture these dynamics remains a challenging task. From a theoretical standpoint, evolutionary processes are typically modeled using continuous-time Markov chains, but the immense state space of protein sequences renders direct modeling intractable. To mitigate this computational bottleneck, classical models of sequence evolution assume that sites evolve independently according to a context-agnostic substitution process. While this assumption enables efficient likelihood computation and has underpinned decades of progress in phylogenetics, it comes at the cost of ignoring epistatic interactions between sites. As a consequence, these models have limited expressivity and often produce unrealistic evolutionary trajectories. This limitation has restricted their applicability in antibody sequence design and optimization, especially in comparison to modern antibody language models, which implicitly capture complex intra-sequence dependencies but lack explicit evolutionary grounding.

**Figure 1.**
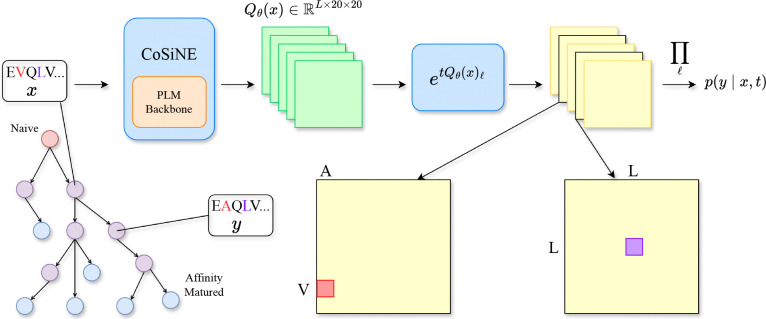
Overview of COSINE. Given an antibody sequence x, the neural network outputs site-specific rate matrices conditioned on the full sequence. Each matrix is evolved for duration t to yield a per-site transition distribution $p\left(y_{l}\;|\;x,t\right)$. Assuming conditional independence, we take the product of the per-site transition probabilities to yield the full sequence transition probability p(y∣x,t).

In this work, we aim to combine the strengths of these two paradigms by introducing a **co**nditionally **s**ite-**i**ndependent **n**eural **e**volution model (COSINE) that learns to simulate antibody affinity maturation while capturing epistatic interactions within the sequence (Figure 1). COSINE uses a neural network to parameterize site-specific rate matrices conditioned on the full sequence context, enabling a factorized transition likelihood that still captures dependencies among sites. In Section 2, we formalize the affinity maturation generative process and describe foundational CTMC theory for sequence evolution models. In Section 3, we review related work. In Section 4, we introduce the COSINE model and prove that it can learn epistatic effects by approximating an expressive sequential point mutation process. We fit COSINE to about a hundred thousand clonal trees and employ a principled Gillespie sampling algorithm to simulate clonal expansion. In Section 4.1, we describe how to disentangle the effects of selection and mutation on the affinity maturation process so that COSINE can be used to infer antibody fitness. In Section 4.2, we propose a classifier guidance procedure to steer the functional properties of antibodies generated by COSINE. In Section 5, we demonstrate the effectiveness of our approach by outperforming existing models on zero-shot variant effect prediction and *in silico* antibody optimization tasks. Finally, in Section 6, we discuss limitations of the current framework and outline directions for future work.

## 2. Background

We consider the evolutionary history of antibody families, where each family f is represented by a clonal tree $T_{f}$. At the root of $T_{f}$ is a naive antibody corresponding to an unmutated sequence derived from genetic recombination. Over time, this naive sequence accumulates somatic mutations, which are recorded in the tree as directed edges (x,y) with an associated branch length $t\in R^{+}$. Informally, we call τ=(x,y,t) an evolutionary transition from the parent x to the child y over time t. Following the standard in phylogenetics, we assume that t is calibrated to the expected number of mutations per-site between x and y.

### 2.1. Markov Models of Protein Evolution

Protein sequence evolution is most commonly modeled via continuous-time Markov chains (CTMCs). In general, given a discrete state space 𝒮, a CTMC is completely defined by an initial distribution $p_{0}$ and a rate matrix $Q\in R^{\left|𝒮|\times |𝒮\right|}$. In fact, Q is all we need to compute the transition probability

(1) $$p(y|x,t):=exp(tQ{)}_{xy}$$

for maximum-likelihood estimation of Q as well as for most interesting sampling tasks. Unfortunately, tractability is limited by the matrix exponential exp(tQ), which costs $O(|𝒮{|}^{3})$ time. For protein sequences of length L with the typical 20 amino acid vocabulary 𝒜, this step is problematic for even L>2 since $|S|=|𝒜{|}^{L}=20^{L}$.

### 2.2. Classical “Independent-Sites” Models

To circumvent this issue, classical models of protein evolution make the independent-sites assumption to enable the factorization of the full sequence Markov chain into L independent chains, one for each site of the protein. Thus, instead of inferring a sequence-level transition matrix $Q\in R^{|𝒜{|}^{L}\times |𝒜{|}^{L}}$, classical models infer $Q_{\ell }\in R^{\left|𝒜|\times |𝒜\right|}$ for ℓ∈{1,…,L}. The resulting transition probability is now tractable with a time complexity of $O(L|𝒜{|}^{3})$:

$$p(y|x,t):=\prod_{\ell =1}^{L}exp{\left(tQ_{\ell }\right)}_{x_{\ell },y_{\ell }}$$

However, the independent-sites assumption leads to model misspecification because it fully ignores higher-order epistatic effects between sites. In fact, popular classical models like WAG (Whelan & Goldman, 2001) and LG (Le & Gascuel, 2008) learn a single $Q\in R^{\left|𝒜|\times |𝒜\right|}$ shared by all L sites, with an optional scaling factor $Q_{\ell }=\alpha_{\ell }Q$ to support site rate variation. As one can imagine, these models are under-expressive and fail to sample high quality proteins over long branch lengths. As such, their application in protein design and optimization has been limited.

### 2.3. Sequential Point Mutation Process

To capture epistatic effects, the class of Markov chains used to model protein evolution is commonly assumed to be a sequential point mutation process (Pedersen & Jensen, 2001; Gesell & von Haeseler, 2006). In this regime, we assume that mutations in the sequence are always introduced one at a time, prohibiting simultaneous events at multiple sites. This introduces a sparse structure to the rate matrix since its entries $Q_{xy}$ can only be non-zero if x and y differ by a Hamming distance of 1. While this model ignores insertions and deletions (indels), it is a standard assumption in protein evolution that is particularly well-suited for antibodies. This is because somatic hypermutation is biologically driven by single-nucleotide substitutions (Noia & Neuberger, 2007), while indels are rare and typically filtered out by purifying selection to preserve antibody structure (Wu et al., 2003). We denote the space of such matrices by $Q\in R^{|𝒜{|}^{L}\times |𝒜{|}^{L}}$.

## 3. Related Work

To address the lack of site heterogeneity in classical models, Le et al. (2012) developed a mixture model that partitions sites into 4 rate categories and estimates a different substitution matrix for each partition. In the extreme case, Prillo et al. (2024) estimated a separate rate matrix for each site. Their model, SiteRM, achieved strong variant effect prediction results, outperforming many protein language models on the ProteinGym benchmark (Notin et al., 2023).

To move beyond the independent-sites assumption, a common approach consists of conditioning the parameterization of site specific mutation rates on the surrounding sequence context. This idea was first explored by Felsenstein & Churchill (1996) who used a Hidden Markov Model to assign per-site rate categories depending on adjacent sites. Bérard et al. (2008) proposed a neighbor-dependent DNA substitution model that adds extra terms to a baseline rate matrix in the presence of CpG sites. More recently, Albors et al. (2025) used a neural network that takes an entire DNA sequence as input and outputs the parameters of an F81 model (Felsenstein, 2005) for the central position of this sequence.

In the domain of antibody evolution, models must also account for the inherent biases of somatic hypermutation (SHM). Recently, the Deep Amino acid Selection Model (DASM) demonstrated a method for disentangling these mutational biases from functional selection (Matsen IV et al., 2025). However, the DASM transition likelihood requires a manual clamping of selection scores to ensure that the resulting likelihoods represent a valid probability distribution. In contrast, COSINE derives a selection score through a log-likelihood ratio between a neural CTMC and a pre-trained SHM model (Sung et al., 2025). This formulation ensures mathematical consistency without heuristic constraints, enabling COSINE to model the affinity maturation process with more accuracy. We provide a detailed comparison of our model against DASM in Section A.3.

## 4. COSINE: Conditionally Site-Independent Neural Evolution Model

To address the limitations of classical models, we developed the COSINE model, which introduces two key modifications that mitigate model misspecification and leverage high-dimensional neural parameterization. First, we decouple rate estimation by learning site-specific rate matrices $Q_{\ell }$ for each site ℓ instead of a single unified matrix with scaling factors. Second, we model each evolutionary transition τ=(x,y,t) with its own set of rate matrices ${\left\{Q_{\ell }^{\left(\tau \right)}\right\}}_{\ell -1}^{L}$, which are inferred by conditioning on the parent sequence x. Concretely, the transition probability of COSINE is given by

(2) $$p_{\theta }(y|x,t)=\prod_{\ell =1}^{L}exp{\left(tQ_{\theta }(x{)}_{\ell }\right)}_{x_{\ell },y_{\ell }}$$

where $Q_{\theta }:R^{|𝒜{|}^{L}}\to R^{L\times |𝒜|\times |𝒜|}$ is a function parameterized by a neural network. Intuitively, COSINE relaxes the standard independent-sites assumption by estimating state-conditional rates, which enables the model to learn epistatic effects. In the next section, we provide theoretical grounding for COSINE by showing that our model constitutes a first-order approximation of a sequential point mutation process over the full sequence space. We further justify this framework by establishing an upper bound for the error between the transition probability of our model and the transition probability of the point mutation process $P(y|x,t)=exp(tQ{)}_{xy}$. Finally, we propose to sample from COSINE using a Gillespie procedure that provably samples from P(y∣x,t) under certain conditions.

### COSINE is a First-Order Approximation of Q.

Let $Q\in R^{|𝒜{|}^{L}\times |𝒜{|}^{L}}$ be the generator rate matrix of a sequential point mutation process over protein sequences of length L with amino acid vocabulary 𝒜. The vector of transition probabilities to other states after some time t is given by indexing the transition matrix as follows:

$$P(\cdot |x,t)={\left(e^{tQ}\right)}_{x_{,\cdot }}\in R^{|𝒜{|}^{L}}$$

#### Proposition 4.1.

(***Proof in*** Section D.1) *Assume the per-site rate matrices* $Q_{\theta }(x{)}_{\ell }$ *are parameterized such that*

$${\left(Q_{\theta }(x)\ell \right)}_{x_{\ell },y_{\ell }}=Q_{x,y}$$

*for all* x,y *with Hamming distance* d(x,y)=1 *and* ℓ *is the unique site where* x *and* y *differ. Then, the error between the transition probability vectors is bounded such that*

(3) $${\left\|P(\cdot |x,t)-p_{\theta }(\cdot |x,t)\right\|}_{1}\leq (\lambda t{)}^{2}=O\left(t^{2}\right)$$

where $\lambda ={max}_{x}\left\{-Q_{x,x}\right\}$ is the maximum exit rate of any given state.

Intuitively, the theorem shows that our model offers a principled way of capturing first-order mutation dynamics while restricting the approximation error to epistatic effects that grow quadratically in the branch length. Moreover, this approximation is particularly well-suited for affinity maturation because high-frequency clonal selection typically constrains lineages to short branch lengths. In this regime, the first-order evolutionary signal captured by our model O(λt) dominates the quadratic approximation error $O\left(\lambda^{2}t^{2}\right)$.

#### Gillespie Sampling with COSINE.

Although the factorized transition probability in Equation (2) allows for trivial sampling, it incurs an error with respect to the more expressive sequential point mutation process that scales quadratically with the branch length. To address this limitation, we propose a Gillespie sampling algorithm adapted to the instantaneous rates of the COSINE model. In Lemma 4.2, we show that this procedure provably samples from P(⋅∣x,t) under certain conditions.

#### Lemma 4.2.

(***Proof in*** Section D.2) *Let* $x_{0},\ldots ,x_{t_{N-1}},x_{t_{N}}$ *be the trajectory of sequences sampled from the Gillespie* Algorithm S1, *using branch length* t *and starting sequence* $x_{0}$. *For all* $x\in \left\{x_{0},\ldots ,x_{N-1}\right\}$, *assuming that*

$${\left(Q_{\theta }(x{)}_{\ell }\right)}_{x_{\ell },y_{\ell }}=Q_{x,y}$$

*holds for all sequences y with Hamming distance* d(x,y)=1, *then* $x_{t_{N}}∼P\left(\cdot |x_{0},t\right)$.

The validity of Proposition 4.1 and Lemma 4.2 relies on ${\left(Q_{\theta }{\left(x\right)}_{\ell }\right)}_{x_{\ell },y_{\ell }}$ approximating the instantaneous non-zero rates in the point mutation process rate matrix $Q_{x,y}$. However, because COSINE is trained with the approximate transition probability in Equation (2), its estimated rates will incur some bias due to the marginalization of unobserved intermediate states on long branches. We acknowledge this theoretical limitation and study its effect thoroughly in Section C.1. In practice, we show that the transition probability error of our model indeed scales quadratically with the branch length and that Gillespie sampling produces final state distributions that match those of the true process much more closely (Sections C.2 and C.3).

### 4.1. Inferring Antibody Fitness Landscapes from Affinity Maturation

Beyond accurately simulating affinity maturation, it is of great interest to explicitly learn the fitness of a given antibody sequence to enable the design of antibodies with desirable properties. The fitness of an antibody sequence is the objective that guides the affinity maturation process, which is typically some combination of properties including expressibility, binding affinity for a target epitope, and lack of affinity for self-epitopes. These properties are also desirable for artificially engineered antibodies.

A common method for determining an antibody model’s ability to predict fitness is through zero-shot Variant Effect Prediction (VEP) with Deep Mutational Scanning (DMS) assays. In these assays, a library of mutants is produced from a wildtype antibody sequence, and each mutant is evaluated for a certain property, such as binding affinity for a specific epitope or level of expression.

For antibody language models that learn the marginal distribution of sequences p(x), the most common approach to evaluating on DMS datasets is to correlate the sequence perplexity from the model with the true fitness of the sequence, under the assumption that lower perplexity (corresponding to higher predicted likelihood) corresponds with higher fitness. COSINE, however, learns a conditional distribution p(y∣x,t), requiring a different approach to DMS evaluation.

#### Somatic Hypermutation Models.

Molecular evolution, including somatic evolution processes such as affinity maturation, can be viewed as a two-step process: First, mutations are introduced by an underlying mutational mechanism. Second, these mutations are filtered by selection according to the fitness advantages or disadvantages they confer.

It is generally believed that mutations arise independently of natural selection. During antibody affinity maturation, this underlying process is known as somatic hypermutation (SHM). Machine learning models, such as Thrifty (Sung et al., 2025), have been developed to predict rates of SHM from surrounding context by training on sequences that have undergone frameshift mutations, meaning they are under little or no selection, preventing any confounding.

#### Disentangling Selection from SHM.

To estimate the fitness landscape, we treat the underlying SHM process as the baseline. Following the mutation-selection framework introduced by Halpern & Bruno (1998), we decompose $Q_{xy}$, the observed transition rate from sequence x to sequence y, as:

(4) $$Q_{xy}=k\;\mu_{xy}\;P_{fix}(x\to y),$$

where $\mu_{xy}$ is the transition rate under SHM, $P_{\text{fix}}(x\to y)$ is the probability of fixation, and k is an arbitrary scalar.

Using the small t approximation $p_{\theta }(y|x,t)=exp{\left(tQ_{\theta }\right)}_{xy}\approx t{\left(Q_{\theta }\right)}_{xy}$for x≠y, we can manipulate Equation (4) to derive the following *selection score* that we use to evaluate COSINE on DMS assays:

(5) $$Score(x\to y)\;=logp_{\theta }(y|x,t)-logq(y|x,t)\;\;\approx log\left(P_{\text{fix}\;}(x\to y)\right)+C,$$

where q(y∣x,t) is the probability of sequence x mutating to y under Thrifty (more details on how this is calculated in Section B.3.2) and $p_{\theta }(y|x,t)$ is the probability of the transition under COSINE. In Section D.3, we rely on standard population genetics theory (Kimura, 1962) to show that $P_{\text{fix}}(x\to y)$ is monotonic with respect to $s_{xy}$, the selective advantage of allele y over allele x.

### 4.2. Conditional Sequence Optimization via Guided Gillespie Sampling

Biologists often wish to design antibodies that strongly bind to a target of interest while maintaining expressibility and stability in the human body. While we are able to show that our model learns a very strong prior for the latter properties, there remains a performance gap for target-specific properties such as binding affinity. Indeed, given that the antigens associated with the transitions in our dataset are unknown, it is possible that the selection scores inferred by our model (Equation (5)) do not align with wet lab measurements for binding affinity to an arbitrary target. Instead of fine-tuning COSINE with additional expensive experimental data, we propose a classifier guidance approach to sample from the desired posterior transition density p(y∣x,t,z) conditioned on the antigen of interest z.

Nisonoff et al. (2025) showed that as we take the limit of t→0, the above posterior transition density is completely defined by the following generator rate matrix:

(6) $${\left(Q_{z}^{\left(\gamma \right)}\right)}_{x,y}={\left[\frac{p(z\;|\;y)}{p(z\;|\;x)}\right]}^{\gamma }Q_{x,y},$$

where $\gamma \in R^{+}$ is a hyperparameter that controls the guidance strength and p(z∣y) is the marginal likelihood of the antigen z given the selection of antibody y. Learning p(z∣y) for an arbitrary class of high-dimensional antigen sequences is difficult; therefore, we approximate it using a surrogate likelihood for the measured binding affinity between y and z. Specifically, we model the probability of the antigen being z as proportional to the probability that the affinity r exceeds a threshold $r_{0}$. Furthermore, we assume that the affinity r between y and z is normally distributed with mean $\mu_{\theta_{z}}(y)$ and variance $\sigma_{\theta_{z}}^{2}(y)$, such that

(7) $$p(z\;|\;y)\propto P\left(r>r_{0}\;|\;y;\theta_{z}\right)=\Phi \left(\frac{\mu_{\theta_{z}}(y)\;-\;r_{0}}{\sigma_{\theta_{z}}(y)}\right),$$

where Φ denotes the c.d.f. of the standard normal distribution. Instead of fixing a global value for $r_{0}$, which could lead to vanishing guidance weights in regions where sequences are far from the threshold, we select $r_{0}$ adaptively by setting it to the parent’s mean prediction $r_{0}=\mu_{\theta_{z}}(x)$. Under the assumption of a symmetric predictive distribution (like the Gaussian used here), the denominator $P\left(r>\mu_{\theta_{z}}(x)\;|\;x,\theta_{z}\right)$ is exactly 1/2. Consequently, Equation (6) becomes

(8) $${\left(Q_{z}^{\left(\gamma \right)}\right)}_{x,y}={\left[2\cdot \Phi \left(\frac{\mu_{\theta_{z}}(y)\;-\;\mu_{\theta_{z}}(x)}{\sigma_{\theta_{z}}(y)}\right)\right]}^{\gamma }Q_{x,y}.$$

In its current form, Equation (8) is very expensive to compute since we need to query $\mu_{\theta_{z}}(y)$ and $\sigma_{\theta_{z}}(y)$ for all sequences y with Hamming distance d(x,y)=1, which corresponds to L×(|𝒜|-1)+1 calls to the predictor for each x. Instead, we employ a first-order Taylor series approximation about $\mu_{\theta_{z}}(x)$ while assuming a locally constant variance: $\sigma_{\theta_{z}}(y)\approx \sigma_{\theta_{z}}(x)$. By linearizing the mean as $\mu_{\theta_{z}}(y)\approx \mu_{\theta_{z}}(x)+\nabla_{x}\mu_{\theta_{z}}(x{)}^{⊤}(y-x)$, we can estimate the change in fitness for all y via a single gradient computation. Substituting these approximations into Equation (8) yields the Taylor-approximated guidance (TAG) form:

(9) $${\left(Q_{z}^{\left(\gamma \right)}\right)}_{x,y}\approx {\left[2\cdot \Phi \left(\frac{\nabla_{x}\mu_{\theta_{z}}(x{)}^{⊤}(y\;-\;x)}{\sigma_{\theta_{z}}(x)}\right)\right]}^{\gamma }Q_{x,y}.$$

We apply TAG to Algorithm S1 to obtain *Guided Gillespie* sampling and provide the pseudocode in Algorithm S2. Although *Guided Gillespie* follows from Nisonoff et al., our formulation is applied to the rate matrix Q of any sequential point mutation process, which is not constrained by boundary time conditions like the generators of flow matching or discrete diffusion models. Notably, this implies that our predictor does not need to be trained on noisy sequences, enabling the straightforward application of sequence-to-property predictors trained naively on experimental data.

## 5. Experiments

### 5.1. Fitting COSINE on a Clonal Tree Dataset

We fit COSINE on a dataset of ∼2 million evolutionary transitions constructed from 5 public sources (Jaffe et al., 2022; Tang et al., 2022; Vergani et al., 2017; Engelbrecht et al., 2025; Rodriguez et al., 2023), with train and test splits that match those of the DASM model (Matsen IV et al., 2025). We initialized COSINE from the 150M parameter ESM2 checkpoint (Lin et al., 2023) and replaced the language modeling head with a randomly initialized output head that uses the softplus activation function to estimate the offdiagonal rates of $Q_{\theta }(x)\ell$. To satisfy the properties of a valid rate matrix, we set the diagonal entries of $Q_{\theta }(x{)}_{\ell }$ to the negative sum of their respective rows. We also inserted a chain-break token between the heavy and light sequences of paired antibodies, enabling simultaneous reasoning over both chains. We performed end-to-end training of all parameters using a polynomial decay learning rate schedule and early stopping based on validation loss (Additional details in Appendix A).

**Figure 2.**
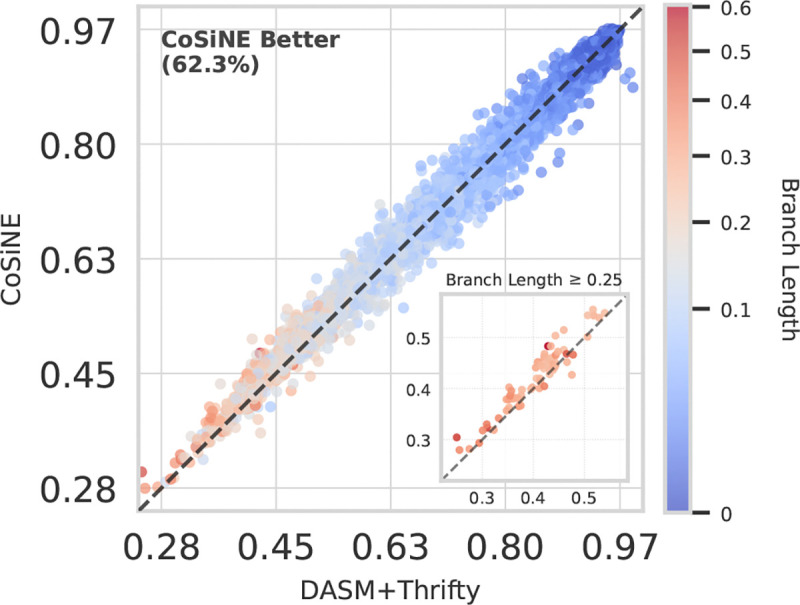
Mean per-site likelihood of COSINE versus DASM+Thrifty on held out evolutionary transitions from the test set. COSINE achieves better model fit, especially on transitions with longer branch lengths (*t* ≥ 0.25).

We found that COSINE fits this data remarkably well, achieving a test perplexity of 1.264 for heavy chain transitions in the Rodriguez et al. dataset (see Appendix A for our perplexity definition). In a head-to-head comparison with DASM+Thrifty, COSINE fits the test transitions with greater per-site likelihood in 62.3% of cases (Figure 2). Interestingly, this improvement is even more significant for long branch lengths (t>0.25).

### 5.2. COSINE Captures Intra- and Inter-Chain Epistasis

**Figure 3.**
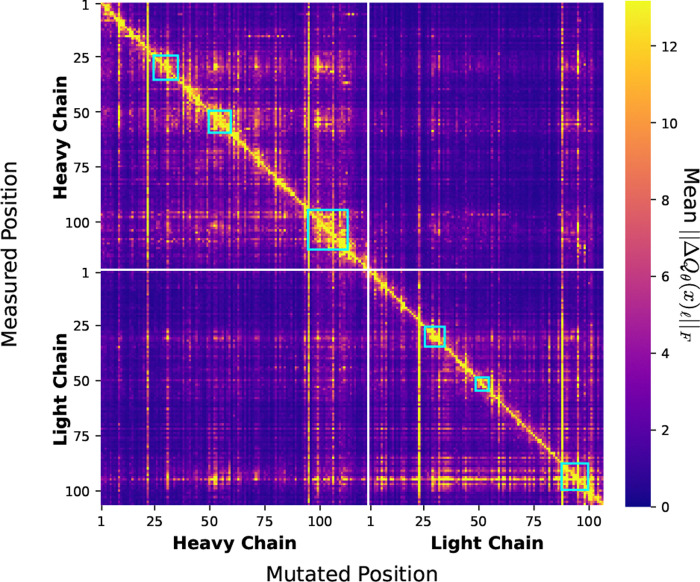
Categorical Jacobian for antibody 47D11 from CoVAbDab. The heatmap displays the sensitivity of the model’s output predictions (y-axis) to specific mutations in the input sequence (*x*-axis). Sensitivity is measured as the Frobenius norm of the change in predicted rate matrix, averaged over all possible mutations. **Cyan squares** denote the CDR regions for both chains.

To investigate the epistatic effects learned by COSINE, we calculate the categorical Jacobian (Figure 3), inspired by Zhang et al. (2024), for an arbitrary antibody sequence from the CoV-AbDab database (Raybould et al., 2020). Specifically, for each possible single amino acid substitution in the input sequence, we measure the change in the output at all positions (See Algorithm S3).

The majority of the measured effect is found along the diagonal. This is expected since changes in a given site and its immediate neighbors will likely have a major impact on its rate matrix due to the importance of local interactions. However, there is a considerable amount of off-diagonal activity as well. For example, we see that within the CDR regions, denoted by cyan squares, there is a high degree of off-diagonal coupling. This is biologically plausible, as the CDR regions combine to form the antigen-binding pocket of the antibody, so changes in one CDR residue are often correlated with changes in other CDR residues to preserve binding geometries. In fact, we see significant changes in the predictions at CDR1 and CDR2 caused by mutations in CDR3 within both the heavy and light chains. COSINE also detects this dependence across chains. For example, when introducing mutations to light chain CDR3, three distinct sensitivity hotspots are induced in the heavy chain associated with the three CDR regions.

### 5.3. Zero-Shot Variant Effect Prediction

We evaluate COSINE’s performance on DMS assays by measuring the Spearman correlation of our selection score (Equation (5)) with the experimental fitness values. To calculate the score, we set the parent x to be the wildtype sequence from which the mutants in the assay are derived. We fix t=0.2 for all assays and VEP experiments.

Evaluation is performed on four DMS assays, two measuring expression and two measuring binding, taken from the FLAb2 benchmark (Chungyoun & Gray, 2025). These assays were selected for their large sample sizes of sequences with the same length in an attempt to limit spurious correlations. As baselines, we compare against AbLang-2, DASM, ProGen2 Small, ProGen2 Medium (best performing model for all FLAb2 binding datasets), ESM-2 150M (best performing model for all FLAb2 expression datasets), and ESM-2 650M (Olsen et al., 2024b; Matsen IV et al., 2025; Nijkamp et al., 2022; Lin et al., 2023). Further details on the DMS assays and evaluation with baseline models are provided in Section B.3.1.

The VEP results are shown in Table 1, where COSINE outperforms all other baselines on all datasets except Koenig Expression Light Chain, where it falls short of ProGen2 Small by 0.005. Interestingly, COSINE substantially outperforms all other models on the Adams dataset, where the wildtype sequence is a mouse antibody. Although COSINE has never been trained on mouse antibodies, we suspect that the pretrained ESM2 backbone helps with generalization.

**Figure 4.**
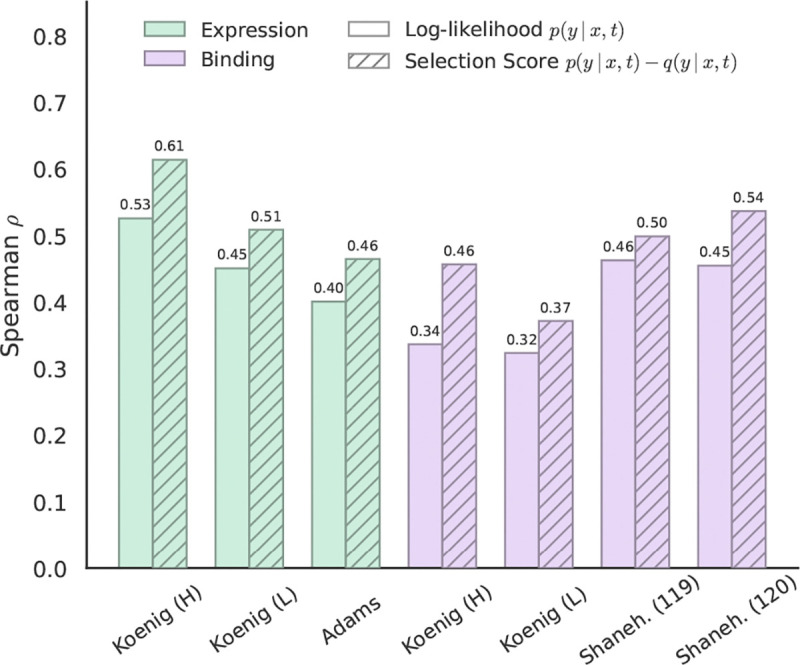
DMS evaluation results for COSINE across expression (green) and binding (purple) assays. A solid color indicates the log-likelihood, $log\;p_{\theta }(y\;|\;x,t)$, and hatching indicates the selection score defined in Equation (5), which utilizes Thrifty likelihoods to separate selection from neutral mutation.

We investigate the efficacy of our approach to correcting for SHM on zero-shot VEP performance. As shown in Figure 4, our selection score leads to increased correlation with fitness across all datasets. Figure S6 provides an intuitive picture of how the SHM correction helps isolate a selection signal from COSINE. We also see that the ability to model interchain interactions is important. In Section C.6, we ablate this ability by passing in just the heavy or light chain alone, finding that the selection score’s correlation with fitness drops substantially for some datasets.

### 5.4. Guided Affinity Maturation from Naive Antibodies

We demonstrate the potential of COSINE to simulate affinity maturation towards high-affinity binders, starting from naive antibody sequences. We used predictors from (Jin et al., 2021) trained on the CoV-AbDab database, which employ an RNN encoder to transform heavy chain sequences into neutralization scores $\mu_{\theta_{z}}$ against the SARS-CoV-1 and SARS-CoV-2 receptor binding domains. We adopted MC dropout (Gal & Ghahramani, 2016) to estimate $\sigma_{\theta_{z}}$. From here, we randomly picked naive sequences from the OAS database (Olsen et al., 2022a) and recursively sampled down the tree in Figure S1 using *Guided Gillespie*.

**Figure 5.**
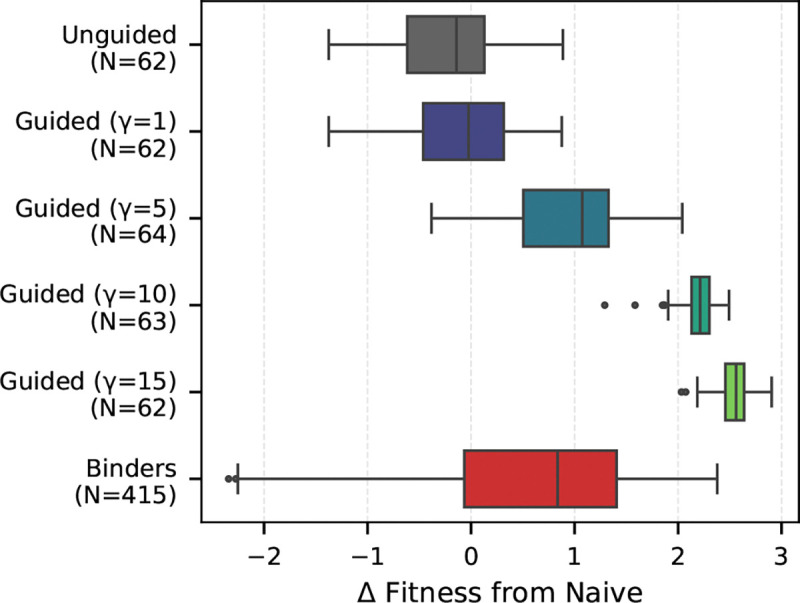
*Guided Gillespie* consistently steers the predicted binding affinity against SARS-CoV-1 of the sampled leaf sequences. We plot the change in predicted binding affinity from the naive root sequence used to start sampling. Known binders from CoV-AbDab are plotted for reference in red.

We compare the affinity gain of our generated leaf sequences against 415 SARS-CoV-1 and 766 SARS-CoV-2 binders from the Cov-AbDab database. As shown in Figure 5, unguided sampling yields a distribution centered near zero for the SARS-CoV-1 target, confirming that the base model is unbiased regarding antigen specificity. Introducing guidance consistently shifts this distribution towards higher affinity. While γ≥10 produced scores exceeding biological binders (likely exploiting oracle uncertainty), we noticed that γ=5 generates affinity profiles that overlap with the real binders.

**Figure 6.**
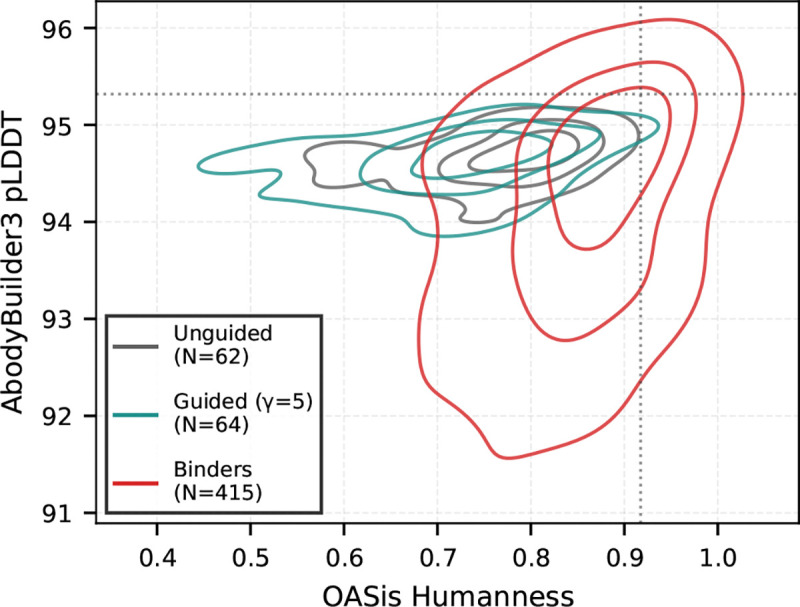
Antibodies sampled by COSINE under guidance (γ=5) maintain high structural quality (AbodyBuilder3 pLDDT) and humanness (OASis). We compare against unguided leaf samples (black) and CoV-AbDab binders (red).

We therefore adopted γ=5 for further evaluation. Figure 6 demonstrates that these guided samples maintain structural plausibility (AbodyBuilder3 pLDDT, Kenlay et al. (2024a)) and humanness (OASis, Prihoda et al. (2021)) comparable to both unguided and natural sequences. These results hold across both targets and many naive seed sequences (Section C.9), underscoring the generalizability of our approach.

### 5.5. Local Optimization of Antibody CDRs

Beyond simulating affinity maturation over long branch lengths, we evaluated whether COSINE could perform constrained local optimization. We tasked the model with refining a SARS-CoV-1 binder using a strict budget of five mutations within the CDR regions. Instead of conditioning on a particular branch length, we fixed the number of Gillespie steps taken, which ensures that the mutation ceiling is respected. We benchmarked against a genetic algorithm (GA) and a Product-of-Experts (PoE) sampler inspired by Gordon et al. (2024) that steers protein language models (ESM-2 and AbLang-1) using the oracle. Among these methods, COSINE achieved the highest gains in predicted binding affinity while maintaining high humanness (Table S4). A full description of this experiment is provided in Section B.5.

## 6. Conclusion and Limitations

In this work, we presented COSINE, a method that reconciles the expressivity of deep protein language models with the temporal dynamics of phylogenetic substitution models to effectively model the process of antibody affinity maturation. We introduced a mathematically grounded framework for the inference of sequential point mutation CTMCs via a first-order approximation of the true transition likelihood with bounded error. Empirically, we demonstrated the utility of COSINE by achieving state-of-the-art results in antibody variant effect prediction. Furthermore, to our knowledge, we are the first to draw an explicit connection between discrete diffusion and classical sequence evolution models, enabling the application of classifier guidance to steer the sampling process of our model. Our results demonstrate that it is possible to capture both time-dependent evolution and epistatic interactions, leading to a new paradigm for protein sequence design that is grounded in molecular evolution.

Despite these contributions, our approach has limitations that present directions for future work. First, our reliance on a first-order approximation of the CTMC over the full sequence state space leads to model misspecification. Second, our framework currently ignores insertions and deletions, restricting COSINE to equal-length sequences or multiple sequence alignments. Although these limitations are less worrisome for modeling antibody affinity maturation, where evolution is rapid and indels are scarce, they likely present a greater challenge when generalizing to all types of proteins. Nonetheless, we believe COSINE provides a strong foundation for future research into dynamic and expressive generative models of protein evolution.

## Supplementary Material

Supplement 1

## Figures and Tables

**Table 1.** Comparison of deep protein models on VEP benchmarks across expression and binding landscapes as measured by Spearman correlation. The Koenig assays are split depending on which chain is mutated, denoted by (H) and (L) for the heavy and light chains respectively. To maintain the same sequence length across samples, the Shanehsazzadeh (Shaneh.) assay is split into two datasets with heavy chains of lengths 119 and 120. Best performing models are shown in **bold**; second-best are underlined.

Model	Expression			Binding			
	
	Koenig (H)	Koenig (L)	Adams	Koenig (H)	Koenig (L)	Shaneh. (119)	Shaneh. (120)

AbLang-2	0.096	−0.127	−0.097	−0.090	−0.011	0.253	0.209
DASM	0.596	0.474	0.270	0.415	0.327	0.450	**0.536**
ESM2–150M	0.413	0.485	−0.112	0.112	0.266	0.236	0.205
ESM2–650M	0.326	0.429	0.124	0.063	0.265	0.227	0.360
ProGen2-Small	0.407	**0.513**	−0.024	0.098	0.332	0.119	0.070
ProGen-Medium	0.392	0.408	0.231	0.085	0.235	0.299	0.319
CoSine (ours)	**0.613**	0.508	**0.464**	**0.456**	**0.371**	**0.498**	**0.536**

## Impact Statement

This work aims to advance the field of machine learning by developing principled models for learning and simulating biological sequence evolution. By bridging modern deep learning with classical evolutionary modeling, our approach contributes new tools for understanding antibody affinity maturation and, more broadly, molecular evolution. Potential positive impacts include improved computational methods for studying immune responses, informing antibody engineering, and supporting downstream applications in biomedical research such as vaccine design and therapeutic antibody development. These advances could ultimately contribute to better diagnostics and treatments. We view this work as a methodological contribution to machine learning and computational biology, with societal implications that are consistent with and comparable to prior advances in biological sequence modeling.
