# Supplementary material for: Conditionally Site-Independent Neural Evolution of Antibody Sequences
Source: ArXiv. 2026 Mar 15:arXiv:2602.18982v3. Preprint. [Version 3] (PMC12976922)
Supplement: Supplementary file 1 [file NIHPP2602.18982v3-supplement-1.pdf]

A. Additional Details of CoSiNE

A.1. Data Collection and Model Training

To train the CoSiNE model, we compiled B-cell receptor (BCR) sequencing datasets from five sources (Jaffe et al., 2022; Tang et al., 2022; Vergani et al., 2017; Engelbrecht et al., 2025; Rodriguez et al., 2023). We adopted the data processing and phylogenetic inference protocol described by (Matsen IV et al., 2025). Briefly, sequences were clustered into clonal families and naive germlines were inferred using `partis` (Ralph & Matsen IV, 2022). We retained families with at least two productive sequences, defining productivity by the absence of stop codons and the presence of canonical cysteine and tryptophan codons flanking the CDR3 in the same reading frame as the V segment start. Consistent with recent large language model training pipelines (e.g., AbLang-2), we further excluded sequences with mutated conserved signature cysteines. Insertions and deletions were reversed to align all sequences to their naive ancestor without gaps.

For phylogenetic reconstruction, we performed tree inference and ancestral sequence reconstruction (ASR) using IQ-TREE (Nguyen et al., 2014) under the K80 substitution model, using the naive sequence as an outgroup. We accounted for mutation rate heterogeneity across sites via a 4-category FreeRate model. For paired heavy and light chain data, we specifically employed the edge-linked-proportional partition model to allow the chains to evolve at distinct overall rates. This pipeline yielded a final training set of parent-child pairs (PCPs) extracted from the edges of the resulting phylogenetic trees, comprising approximately $\sim$2 million transitions from $\sim$120,000 clonal families collected from 555 individual donors. We defer further details to the Matsen IV et al. paper.

Models were trained with mixed precision (BF16) for a maximum of 1 million steps, using a batch size of 16 with gradient accumulation over 3 steps. We employed the AdamW optimizer with a learning rate of 2.5×10^{-4}, utilizing 5,000 warmup steps followed by a polynomial decay schedule with a power of 2.0. To prevent overfitting, we applied a weight decay of 0.01 specifically to parameters with two or more dimensions (e.g., weights and embeddings), while excluding biases and layer normalization parameters. Gradients were clipped at a norm of 1.0, and training employed early stopping with a patience of 50 intervals based on validation loss. In practice, the loss converged after about 1 day on a single A100 GPU.

A.2. Gillespie and *Guided Gillespie* Sampling

We provide the pseudocode for both unconditional Gillespie sampling and guided Gillespie sampling using CoSiNE. Differences for the guided version are highlighted in red. Notice that only a single evaluation of the predictor is required per step.

Algorithm S1 Gillespie Sampling

input Model Q_θ, start sequence x, branch length t
output Samples $y \sim P(\cdot \mid x, t)$

$t' \leftarrow 0$
while $t' < t$ **do**
  $\lambda_x \leftarrow -\sum_{\ell=1}^{L} Q_\theta(x)_{x_\ell, x_\ell}$
  $\tau \sim \text{Exp}(\lambda_x)$
  if $t' + \tau > t$ **then**
    Return $y \leftarrow x$
  end if
  Sample (ℓ^*, a^*) with $P(\ell, a) = (Q_\theta(x)_\ell)_{x_\ell, a} / \lambda_x$
  $x_{\ell^*} \leftarrow a^*$
  $t' \leftarrow t' + \tau$
end while
Return $y \leftarrow x$

Algorithm S2 Guided Gillespie Sampling

input Model Q_θ, start sequence x, time t, predictor $\mu_\theta, \sigma_\theta$, scale γ
output Samples $y \sim P(\cdot \mid x, t, z)$

$t' \leftarrow 0$
while $t' < t$ **do**
  $g \leftarrow \nabla_x \mu_\theta(x)$
  $\tilde{Q}_{x,y} \leftarrow Q_{x,y} \cdot [2\Phi(g^\top (y - x)/\sigma_\theta(x))]^\gamma$
  $\lambda_x \leftarrow \sum_{y \neq x} \tilde{Q}_{x,y}$
  $\tau \sim \text{Exp}(\lambda_x)$
  if $t' + \tau > t$ **then**
    Return $y \leftarrow x$
  end if
  Sample (ℓ^*, a^*) with $P(\ell, a) = (\tilde{Q}_\theta(x)_\ell)_{x_\ell, a} / \lambda_x$
  $x_{\ell^*} \leftarrow a^*$
  $t' \leftarrow t' + \tau$
end while
Return $y \leftarrow x$

A.3. Comparison of CoSiNE against DASM+Thrifty

During training, DASM (Matsen IV et al., 2025) explicitly factorizes the affinity maturation process into the product of a somatic hypermutation (SHM) process $q(y \mid x, t)$, with a selection function $F(y)$. Inferring both q and F simultaneously from data is not possible since there is now an extra degree of freedom. Instead, DASM utilizes a frozen Thrifty SHM model q_ϕ, trained on out-of-frame transitions, and proposes to learn $F_\theta(y)$ via MLE of the observed transitions.

$$p(y \mid x) = \prod_{\ell=1}^{L} p(y_\ell \mid x, t) = \prod_{\ell=1}^{L} q(y_\ell \mid x, t) F_\theta(y_\ell \mid x) \quad \text{s.t.}$$
$$p(x_\ell \mid x, t) = 1 - \sum_{a \in \mathcal{A}} q(a \mid x, t) F_\theta(a \mid x)$$

The second constraint essentially subsumes the normalizing constant into the probability of no-transition. The authors clamp the sum on the right-hand side of the constraint to be less than 1 in order to maintain a valid probability distribution.

Instead of using this formulation, which requires manual clamping of the selection scores, CoSiNE infers $F_\theta(y \mid x)$ at inference time using a log-likelihood ratio between our pre-trained affinity maturation and somatic hypermutation models (Equation (5)).

B. Experimental Details

B.1. Synthetic Experiments on Single Codons

To empirically validate Proposition 4.1 and Lemma 4.2, we utilized a computationally tractable state space that allows for manipulation of the full rate matrix. Specifically, we modeled short DNA sequences with length $L = 3$ over the vocabulary $\mathcal{D} = \{A, G, C, T\}$, thus obtaining a state space that corresponds exactly to the 64 standard codons. To study processes with different levels of epistasis, we constructed ground truth rate matrices using linear interpolation between two extremes:

$$\mathbf{Q}_{\text{true}} = (1 - \varepsilon)\mathbf{Q}_{\text{factorized}} + \varepsilon\mathbf{Q}_{\text{state-dep}}$$

where $\varepsilon \in [0, 1]$ is the epistasis strength parameter. For $\mathbf{Q}_{\text{factorized}}$ (no epistasis), we sampled site-independent base rates: for each site $\ell \in \{1, 2, 3\}$, we drew a 4×4 rate matrix with off-diagonal entries from Uniform$(0, 2)$ and diagonal entries set to the negative row sum. The global 64×64 matrix $\mathbf{Q}_{\text{factorized}}$ assigns rate $r_{\ell,a,b}$ to transitions differing only at site ℓ with substitution $a \to b$. For $\mathbf{Q}_{\text{state-dep}}$ (maximum epistasis), each of the $64 \times 9 = 576$ transitions with a Hamming distance equal to 1 receives an independent rate drawn from Uniform$(0, 2)$. At $\varepsilon = 0$, rates are perfectly factorizable; at $\varepsilon = 1$, every transition is state-dependent.

For each $\mathbf{Q}_{\text{true}}$ that we drew in this way, we generated 2.5M training samples by drawing branch lengths $b \sim \text{Exp}(\lambda = 0.5)$, sampling start states x uniformly, and sampling end states from $P(\cdot \mid x, t) = \exp(t\mathbf{Q}_{\text{true}})_{x,\cdot}$. We compared three estimators: (1) **Full MLE**, which directly parameterizes all 576 transition rates and optimizes via gradient descent on the exact likelihood; (2) **Factorized**, a neural model that outputs context-dependent site-level 4×4 rate matrices and assumes site-independence during training; and (3) **Factorized SNR**, which uses the same architecture as (2) but applies SNR weighting (Section C.1). All models were trained for up to 1000 epochs with Adam (lr $= 0.01$ for factorized models, lr $= 0.1$ for Full MLE) and early stopping (patience=50). We tested $\varepsilon \in \{0, 0.25, 0.5, 0.75, 1.0\}$ with 3 replicates per level, measuring estimation error as the relative difference in Frobenius norm of the estimated and ground truth rate matrices: $\|\mathbf{Q}_{\text{est}} - \mathbf{Q}_{\text{true}}\|_F / \|\mathbf{Q}_{\text{true}}\|_F$.

Using the trained factorized models, we compare Gillespie sampling (Algorithm S1) against per-site matrix exponentiation (Equation (2)). To evaluate each sampling method without sampling noise, we compute exact transition probability distributions analytically. For Gillespie, we achieve this by first reconstructing the full 64×64 estimated rate matrix by querying the model at all 64 states, then computing the transition probability matrix. We compare both methods for the factorized and SNR-weighted models (4 curves total) by measuring KL divergence to the ground truth transition probability across 30 log-spaced branch lengths from $t = 0.01$ to $t = 10.0$, averaged uniformly over all 64 starting states.

B.2. Calculating the Categorical Jacobian via Perturbation

To quantify the epistatic interactions learned by CoSiNE, we approximate the Jacobian by exhaustively computing all single point mutations. This procedure is described in Algorithm S3 with an example output sensitivity matrix depicted in Figure 3.

Algorithm S3 Categorical Jacobian Computation

Input: Antibody sequence x of length L, Vocabulary $\mathcal{A}$ ($|\mathcal{A}| = 20$)
Output: Sensitivity Matrix $\mathbf{S} \in \mathbb{R}^{L \times L}$
Initialize $\mathbf{S} \leftarrow \mathbf{0}_{L \times L}$
Compute wildtype rate matrices: $Q = Q_\theta(x)$
for $i = 1$ **to** L **do**
  for $a \in \mathcal{A}$ **such that** $a \neq \mathbf{x}_i$ **do**
    $x' \leftarrow x$
    $x'_i \leftarrow a$ {Mutate residue at position i to a}
    $Q' \leftarrow Q_\theta(x')$
    for $j = 1$ **to** L **do**
      $\mathbf{S}_{i,j} \leftarrow \mathbf{S}_{i,j} + \|\mathbf{Q}_j - \mathbf{Q}'_j\|_F$ {Calculate shift in output at position j}
    end for
  end for
  $\mathbf{S}_{i,:} \leftarrow \mathbf{S}_{i,:} / (|\mathcal{A}| - 1)$ {Average over all possible mutations}
end for
Return S

B.3. Variant Effect Prediction Details

B.3.1. DMS Assays and Baseline Models

The results shown in Table 1 came from four DMS assays from the FLAb2 benchmark (Chungyoun & Gray, 2025). We provide more details on these datasets in Table S1. Following Matsen IV et al. (2025), we obtain the Koenig and Shanehsazzadeh (Shaneh.) datasets from commit 67738ee (April 17, 2024) of the FLAb Github repository. The Adams dataset is taken from commit 3453aeb (September 1, 2025). The Adams dataset contains multiple fitness measurements per mutant sequence, so we correlate antibody model predictions with the average fitness per mutant during VEP evaluation.

An additional step we must take when calculating the CoSiNE selection score for these assays is determining the underlying nucleotide sequence for the wildtype antibody so that we can calculate the transition likelihood under an SHM model (see Section B.3.2 for more details). For the Koenig and Shaneh. datasets, we use `IMGT/DomainGapAlign` to map the wildtype amino acid sequence to the closest germline V and J genes (Ehrenmann et al., 2010). We then obtain the V- and J-segment nucleotide sequences from HG38 and backtranslate remaining mismatches and junction regions to the codon with the highest frequency in the human genome ((Nakamura et al., 2000)). The Adams dataset uses mouse antibody 4-4-20 scFv as its wildtype, and its nucleotide sequence was obtained from Addgene plasmid pCT302 (Midelfort et al., 2004).

Table S1. **Overview of Deep Mutational Scanning (DMS) Datasets.** *Avg. Subs* denotes the average number of amino acid substitutions per mutant relative to wildtype.

Original Source	Assay Type	Dataset Name	Mutated Chain	Chain Length		Num. Seqs	Avg. Subs
				Heavy	Light		
Koenig et al. (2017)	Expression	Koenig Expression (H)	Heavy	120	108	2261	1
		Koenig Expression (L)	Light	120	108	2014	1
	Binding (VEGF)	Koenig Binding (H)	Heavy	120	108	2261	1
		Koenig Binding (L)	Light	120	108	2014	1
Adams et al. (2016)	Expression	Adams	Heavy	117	112	2803	1.98
Shanehsazzadeh et al. (2023)	Binding (HER2)	Shaneh. (119)	Heavy	119	107	184	5.22
		Shaneh. (120)	Heavy	120	107	201	5.97

We evaluate zero-shot VEP with six other baseline models, which can be divided into three categories based on their architectures and how they are evaluated.

1. Masked language models (**AbLang-2, ESM-2 150M, ESM-2 650M**) are evaluated via pseudo-perplexity in accordance with the FLAb2 benchmark.
2. Autoregressive models (**ProGen2 Small, ProGen2 Medium**) are evaluated via perplexity, also in accordance with the FLAb2 benchmark.
3. The **DASM** model is evaluated by summing its log selection factors as described in Matsen IV et al. (2025).

B.3.2. Calculating Sequence Likelihoods with Thrifty SHM Model

To estimate sequence transition likelihoods under SHM, we use `ThriftyHumV0.2-59-hc-tangshm`, which provides per-codon transition probabilities with a multihit correction described in Matsen IV et al. (2025). Since the Thrifty model operates in the state space of codons (64 states) and CoSiNE operates in the state space of amino acids (20 states), we must sum over all possible codons that could code for the observed alternate allele when calculating sequence likelihoods.

More formally, let x_ℓ denote the amino acid identity at position ℓ in the wildtype sequence for a DMS assay and $c(x_\ell)$ denote its underlying nucleotide codon (see Section B.3.1 for details on how this is determined). Let y_ℓ denote the corresponding amino acid in the mutant sequence y and $C(y_\ell)$ denote the set of possible codons that could code for y_ℓ. We calculate the likelihood of sequence y under Thrifty as

$$q(y \mid x, t) = \prod_{\ell}^{L} q(y_\ell \mid x_\ell, t) \quad \text{where}$$

$$q(y_\ell \mid x_\ell, t) = \begin{cases} q(c(x_\ell) \mid c(x_\ell), t) & \text{if } y_\ell = x_\ell \\ \sum_{c \in C(y_\ell)} q(c \mid c(x_\ell), t) & \text{if } y_\ell \neq x_\ell. \end{cases}$$

B.4. Guided Affinity Maturation from Naive Antibodies

We randomly sampled naive antibodies from a subset of the OAS database (Olsen et al., 2022a) containing only heavy chain sequences with the IgM isotype from human donors. To guide and score the sampled sequences, we utilized the SARS-CoV-1 and SARS-CoV-2 neutralization predictors from Jin et al., with weights downloaded from `https://github.com/wengong-jin/RefineGNN`. These oracles are only trained on heavy chain sequences. To parameterize the variance σ_{θ_z}, we used MC dropout (Gal & Ghahramani, 2016) with 10 fixed masks. For the numerator in Equation (9), we set the model to evaluation mode and computed the gradient of the output $\mu_{\theta_z}(x)$ with respect to the one-hot input sequence x. Known binders were curated from the CoV-AbDab database. We set the naive antibody at the root of the clonal tree in Figure S1 and recursively sampled down its nodes in breadth first order. We repeated this procedure 5 times for each guidance setting and collected the leaf sequences for comparison. The selected tree has 13 leaves, so a non-repetitive sampler would produce 65 unique leaf sequences.

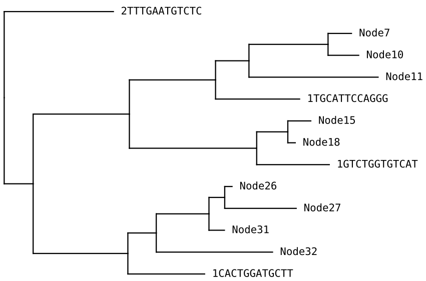

Figure S1. **Clonal Tree used for Guided Affinity Maturation Experiment in Section 5.4**

B.5. Guided Optimization of Antibody CDRs

All methods were tasked with optimizing a SARS-CoV-1 binder from the CovAbDab database. Optimization was restricted to the CDRs, identified via the IMGT numbering scheme (Ehrenmann et al., 2010), with a strict budget of at most 5 mutations from the seed sequence. We generated 1,000 variants per method. We used the same SARS-CoV-1 neutralization predictor as Section B.4. To ensure a fair comparison, all methods (excluding the reference Greedy baseline) were constrained to use ≤ 5 oracle calls per generated sequence. We compared CoSiNE against the following baselines:

Genetic Algorithm (GA). We evolved a population of 1,000 sequences over 9 generations. In each generation, the top 50% of sequences were selected based on fitness, and the remaining 50% were regenerated by applying random mutations to the selected parents. To respect the mutation budget without inefficient rejection sampling, we restrict changes to mutated positions if the budget of 5 mutations has been met. This allows for lateral moves or reversions while satisfying constraints.

Product of Experts (ESM-2 and AbLang-1). Following Gordon et al., we sampled variants using protein language models with a product of experts (PoE) strategy to incorporate oracle guidance. This method uses Gibbs sampling from a joint distribution $P(x) \propto P_{\text{MLM}}(x) \cdot \exp(\lambda F(x))$, where P_{MLM} is a masked language model prior. We evaluated both a general protein language model (ESM-2 150M) and an antibody-specific model (AbLang-1). We used AbLang-1 instead of AbLang-2 because the latter strictly requires paired antibody sequences instead of a single VH chain. We set the guidance strength to $\lambda = 50.0$. To respect the computational budget of ≤ 5 oracle calls per sample, we implemented a caching approximation. Before sampling, we pre-computed the fitness effects of all possible single mutations in CDRs. During the Gibbs sampling process, oracle scores were retrieved from this additive cache rather than re-evaluated using the mutated sequence. This approximates the fitness landscape as locally additive.

Greedy Search. As a performance upper bound, we implemented a stochastic hill-climbing strategy. At each step, the algorithm evaluates all possible single mutations ($L \times 19$ variants), selects the top $K = 15$ candidates by fitness gain, and samples the next step using a softmax over their fitness improvements ($\Delta F/T$, with $T = 1.0$). This process repeats for up to 5 steps. While effective, this method requires orders of magnitude more compute (~2700 oracle calls per sequence) and serves only as a reference for the maximum achievable affinity under the given constraints.

Evaluation Metrics. Performance was evaluated across three axes: **Fitness**, measured by the mean and maximum improvement in predicted binding affinity; **Diversity**, quantified by the average pairwise distance within the generated samples; and **Naturalness**, measured using the OASis score.

C. Supplementary Results

C.1. Estimation Error of $Q_\theta(x)_\ell$ via SNR-Weighted MLE

Under the transition likelihood of the CoSiNE model in Equation (2), maximum likelihood inference of $Q_\theta(x)_\ell$ is performed by minimizing the negative log-likelihood of the observed evolutionary transitions

$$\mathcal{L}(\theta) = -\sum_{\tau=(x,y,t)} \sum_{\ell=1}^{L} \log \exp(tQ_\theta(x)_\ell)_{x_\ell, y_\ell}$$

Unfortunately, directly training with this objective will generally fail to satisfy the assumption in Proposition 4.1, which relies on $(Q_\theta(x)_\ell)_{x_\ell,\cdot}$ approximating the instantaneous non-zero rates in $\mathbf{Q}_{x,\cdot}$. Indeed, on branches with large t, this factorized objective encourages $Q_\theta(x)_\ell$ to learn *effective* rates that account for unobserved epistatic interactions and intermediate states between x and y. This results in a time scale dependent bias where the inferred rates for long branches may diverge from $\mathbf{Q}$.

To mitigate this issue, we sought to leverage the insight that the signal-to-noise (SNR) ratio of our first-order approximation scales as $O(1/t)$. We therefore proposed a SNR-weighted loss function:

$$\mathcal{L}_{SNR}(\theta) = \sum_{\tau=(x,y,t)} \frac{1}{\delta + t} \mathcal{L}(\theta; x, y, t)$$

where $\delta \in [0, 1]$ is a hyper-parameter that controls the magnitude of the weighting. In the synthetic codon experiments (detailed in Section B.1), we were able to show that in a data-rich setting, SNR weighting indeed reduces the estimation error compared to an un-weighted MLE objective, especially at high epistasis levels for the ground truth rate matrix (Figure S2). In addition, we found that setting $\delta = 1 - \varepsilon$ yielded the strongest results, which is intuitive since re-weighting is meant to compensate for epistatic effects in the underlying process.

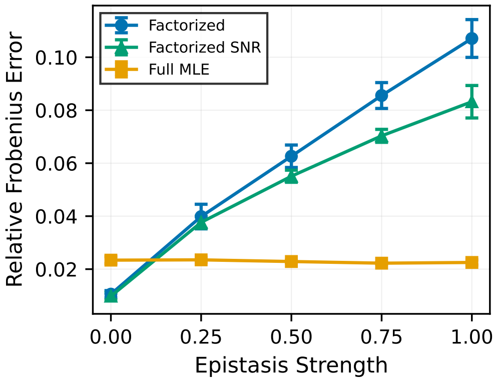

Figure S2. **As ε increases, SNR-weighting (green) reduces the relative Frobenius norm error between the estimated and true rate matrices compared to unweighted MLE (blue), while the Full MLE (yellow) is constant.**

Motivated by this result, we trained CoSiNE on the full clonal dataset using this SNR-weighted objective at different values of $\delta \in \{0.0, 0.5, 1.0\}$. However, we could not identify improvements for these models in comparison to a CoSiNE model trained with the un-weighted loss. We hypothesize that this is due to a relatively data-poor setting for the antibody environment, given that our state space is much larger than the synthetic environment and we have less training data.

C.2. Sampling Error in Synthetic Codon Experiment

Using the synthetic codon environment detailed in Section B.1, we sought to quantify the difference between the sampling distributions of CoSiNE and the true transition probability distribution of the underlying process. For each level of epistasis $\varepsilon \in \{0.0, 0.25, 0.5, 0.75, 1.0\}$, we generated a ground truth rate matrix and trained a CoSiNE model on simulated transitions (see Section B.1). We evaluate performance by measuring the KL divergence between the ground truth transition distribution $P(y \mid x, t)$ and the distributions induced by either Gillespie sampling (Algorithm S1) or the per-site matrix exponentiation approach (Equation (2)).

We found that Gillespie sampling is superior at all epistasis levels and branch lengths. As expected, both sampling methods perform comparably when $\varepsilon = 0$, since the ground truth rate matrix is also site-independent (Figure S3a). However, at $\varepsilon = 1.0$, notice that the KL divergence of the matrix exponential scales quadratically in t before plateauing at a high error, indicating a failure to capture the correct stationary distribution (Figure S3e). In contrast, Gillespie sampling maintains consistently lower divergence and recovers the correct stationary distribution. This is an important result since it empirically validates Proposition 4.1 and supports the claim in Lemma 4.2. As ε increases, we notice that the advantage of Gillespie sampling over the factorized matrix exponential approach also becomes more significant.

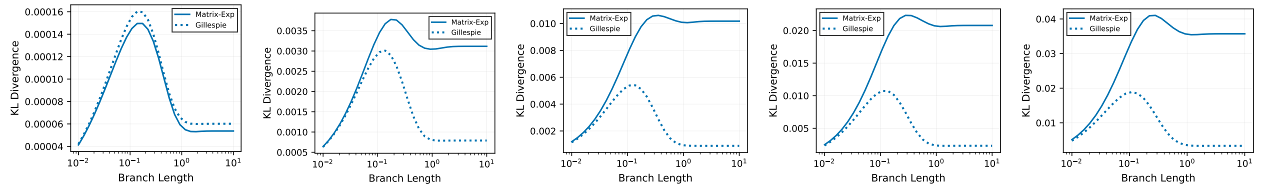

(a) $\varepsilon = 0.00$ *(b)* $\varepsilon = 0.25$ *(c)* $\varepsilon = 0.50$ *(d)* $\varepsilon = 0.75$ *(e)* $\varepsilon = 1.00$

Figure S3. **Gillespie samples are more consistent with the true transition probability distribution than samples from the factorized CoSiNE likelihood at all levels of epistasis.** The difference increases smoothly with the epistasis strength ε in the underlying rate matrix. Ground truth matrix generation and estimation protocols are in Section B.1.

C.3. Sampling Error in Real Antibody Experiment

Although we cannot obtain the transition likelihood for Gillespie samples in the large antibody sequence state space, we sought to validate the synthetic codon results (Section C.2) in the real antibody environment using auxiliary metrics. We selected all clonal trees in the test split with ≥ 4 leaves and sampled new sequences for each tree using the model trained in Section 5.1, conditional on the root sequence. For each leaf node in each tree, we collected the corresponding sampled sequence from both Gillespie and factorized matrix exponential approaches and compared their hamming distance to the real leaf sequence at that node. Assuming that a lower hamming distance to the real leaf sequence indicates lower sampling error, we observe in Figure S4a that leaves simulated with Gillespie sampling are closer to the real leaf sequence in 52.0% of cases, whereas the opposite is true only 38.8% of the time. Next, we computed the hamming distance from the root sequence to every real and simulated leaf sequence. Under the assumption that this distance should be somewhat consistent for corresponding real and simulated leaves, we scatterplot their correlation in Figure S4b for Gillespie samples and Figure S4c for factorized matrix exponential samples. Once again, we find that Gillespie has superior performance, achieving a Pearson correlation of 0.64 while the per-site matrix exponentiation approach only obtains 0.56. Altogether, these results further consolidate our results in the synthetic codon environment and strongly suggest that Gillespie sampling is a principled and superior sampling approach for CoSiNE.

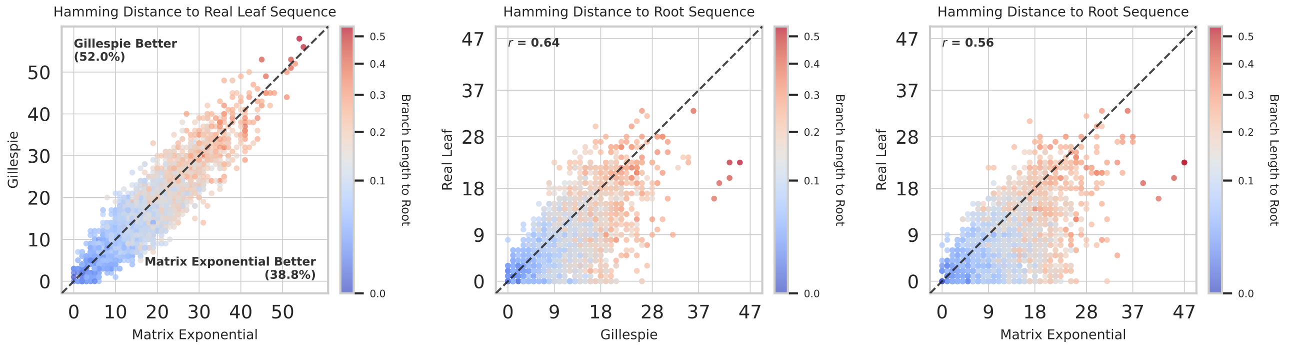

(a) *(b)* *(c)*

Figure S4. **Gillespie sampling exhibits lower sampling error and better preserves evolutionary distances on real antibody clonal families.** a) Comparison of sampling fidelity: Gillespie samples are closer (in Hamming distance) to the true held-out leaf sequence in 48.8% of cases, compared to 40.2% for the factorized matrix exponential. b, c) Correlation between the root-to-leaf Hamming distances of real versus simulated leaf sequences. Gillespie sampling (b) achieves a higher Pearson correlation ($r = 0.62$) with the observed evolutionary distances than the factorized matrix exponential (c) approach ($r = 0.53$).

C.4. Per-Site Entropy Increases with Branch Length

From a randomly selected parent sequence in the test set, we calculated the per-site entropy of the CoSiNE transition likelihood in Equation (2) at different branch lengths (Figure S5). As expected, we found that the entropy increases with the branch length, indicating a larger number of expected mutations. Furthermore, we found that the entropy in CDR regions (red) tends to be higher than the entropy in framework regions (grey).

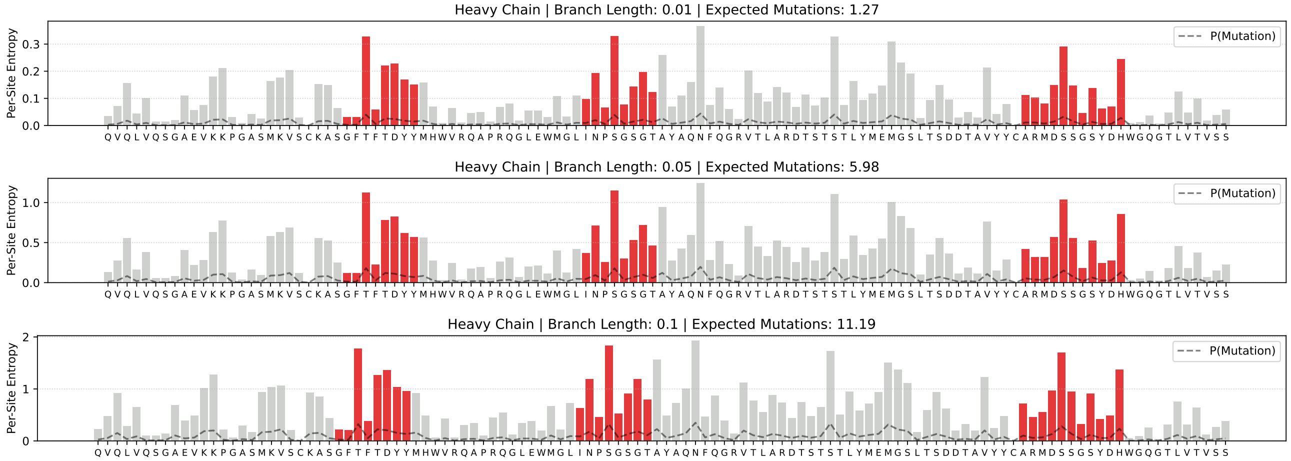

Figure S5. **Per-site entropy across increasing branch lengths.** Entropy profiles for branch lengths 0.01, 0.05, and 0.1 (top to bottom). Red bars denote CDRs. Entropy scales with branch length, showing higher mutational variability in CDRs.

C.5. Log-Likelihood versus Selection Score for DMS Performance

Figure S6 illustrates the effect of our correction method on the Koenig Light Chain expression dataset. In the left plot, there is a clear separation in the data according to the number of nucleotide edits between the wildtype and mutant sequences. This indicates that the model has learned that for a fixed amount of elapsed time, transitions with fewer nucleotide edits are more probable than transitions with more nucleotide edits. This makes sense considering our chosen branch length is somewhat short ($t = 0.2$) and the model is trained to maximize the likelihood of observed transitions. However, if we naively correlate the model's predicted likelihood for the sequence with selection, we introduce the bias that mutations with few nucleotide edits are higher fitness than mutations with more nucleotide edits, which is clearly erroneous. In the plot on the right, we can see that taking the ratio with the likelihood under the Thrifty model removes this edit distance bias as indicated by the fact that the point clouds corresponding to each edit distance now roughly occupy the same space.

The selection score correction does more than just correct for this edit distance bias. For example, certain nucleotide substitutions are more likely to occur than others and without accounting for this variation, those biases will be interpreted as improvements in fitness. In Figure S6 we see that even among mutants with the same nucleotide edit distance, the correlation improves when adding the SHM correction.

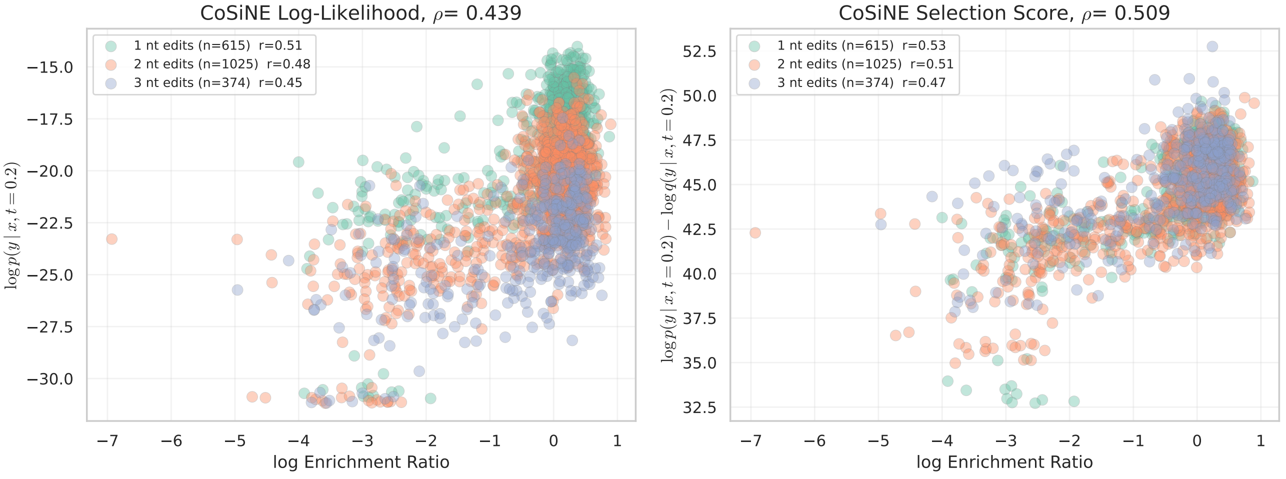

Figure S6. **Analysis of CoSiNE scoring methods against log Enrichment Ratios for the Koenig DMS assay measuring expression (Light Chain). Left**: Log-Likelihood performance ($\rho = 0.439$). **Right**: Selection Score performance ($\rho = 0.509$). Points are colored by the edit distance from wildtype codon to mutant codon. We assume the mutant codon is the one which requires the smallest number of nucleotide edits.

C.6. Additional VEP Experiments

In Table S2 we evaluate CoSiNE on the same datasets as Section 5.3 when conditioning on different amounts of context. Surprisingly, conditioning on just a single chain results in a higher correlation on four out of seven datasets. However, when paired conditioning outperforms, it does so by an average Spearman correlation of **0.121**, compared to just **0.006** when single chain conditioning outperforms. This suggests that inter-chain interactions are crucial for modeling fitness but only under certain contexts. What differentiates these contexts is a direction for future work. It's also worth noting that the majority of the training data for CoSiNE is single chains, suggesting this result could be an artifact of training data composition.

Table S2. **Comparison of CoSiNE on zero-shot VEP with both the heavy and light chains provided as context (Paired) versus just the chain with the mutations (Single).** Blue indicates datasets with mutations on the heavy chain, and red indicates mutations on the light chain.

Model	Expression			Binding			
	Koenig (H)	Koenig (L)	Adams	Koenig (H)	Koenig (L)	Shaneh. (119)	Shaneh. (120)
CoSiNE-Paired	**0.613**	0.508	**0.464**	**0.456**	0.371	0.498	0.536
CoSiNE-Single	0.545	**0.509**	0.234	0.390	**0.375**	**0.504**	**0.549**

In Table S3, we report results from the same experiment as Table 1, but use Pearson correlation instead of Spearman.

Table S3. **Comparison of deep protein models on VEP benchmarks across expression and binding landscapes as measured by Pearson correlation.**

Model	Expression			Binding			
	Koenig (H)	Koenig (L)	Adams	Koenig (H)	Koenig (L)	Shaneh. (119)	Shaneh. (120)
AbLang-2	0.153	−0.109	−0.096	−0.114	−0.108	0.263	0.166
ESM2-150M	0.476	0.539	−0.119	0.044	0.266	0.215	0.197
ESM2-650M	0.384	0.416	0.097	0.009	0.243	0.191	0.308
ProGen2-Small	0.559	0.568	−0.043	0.156	0.276	0.074	0.052
ProGen2-Medium	0.553	0.579	0.209	0.123	0.253	0.296	0.275
DASM	**0.688**	<u>0.674</u>	<u>0.221</u>	<u>0.335</u>	<u>0.316</u>	<u>0.458</u>	<u>0.518</u>
CoSiNE (ours)	<u>0.687</u>	**0.696**	**0.409**	**0.367**	**0.345**	**<u>0.502</u>**	**0.521**

C.7. Taylor-Series Approximated Guidance versus Exact Guidance

To evaluate the utility of our first-order Taylor series approximation for oracle guidance, we compared the fitness improvements and computational costs of exact guidance versus Taylor series approximated guidance. Using the same SARS-CoV-1 oracle as before (Section B.4), we sampled sequences at $t = \{0.01, 0.05, 0.10\}$ with guidance strength $\gamma = 2.0$. We generated 5 samples from each of 3 randomly selected seed antibody sequences, yielding 15 samples per condition.

As shown in Figure S7 (left), both guidance methods produced similar fitness improvements across all branch lengths. Two-sided t-tests confirmed that these differences were not statistically significant at any branch length ($p = 0.571$, $p = 0.918$, and $p = 0.695$), indicating that Taylor approximation does not compromise the quality of *Guided Gillespie* samples.

In contrast, the computational costs differed dramatically between methods (Figure S7, right). Exact guidance requires evaluating the oracle on all single-amino-acid mutants at each Gillespie step, compared to a single call for TAG guidance. This achieves speedups of 488× (0.01), 928× (0.05), and 916× (0.10) across the three branch lengths.

C.8. Local Antibody CDR Optimization Results

Table S4 contains the results discussed in Section 5.5 for the CDR optimization task, with experimental details in Section B.5.

C.9. Guided Affinity Maturation from Additional Naive Antibodies

We provide additional results for the guided affinity maturation sampling experiment using other naive sequences from the OAS database. Notice that in all cases, guidance effectively steers the predicted binding affinity of the generated leaf sequences.

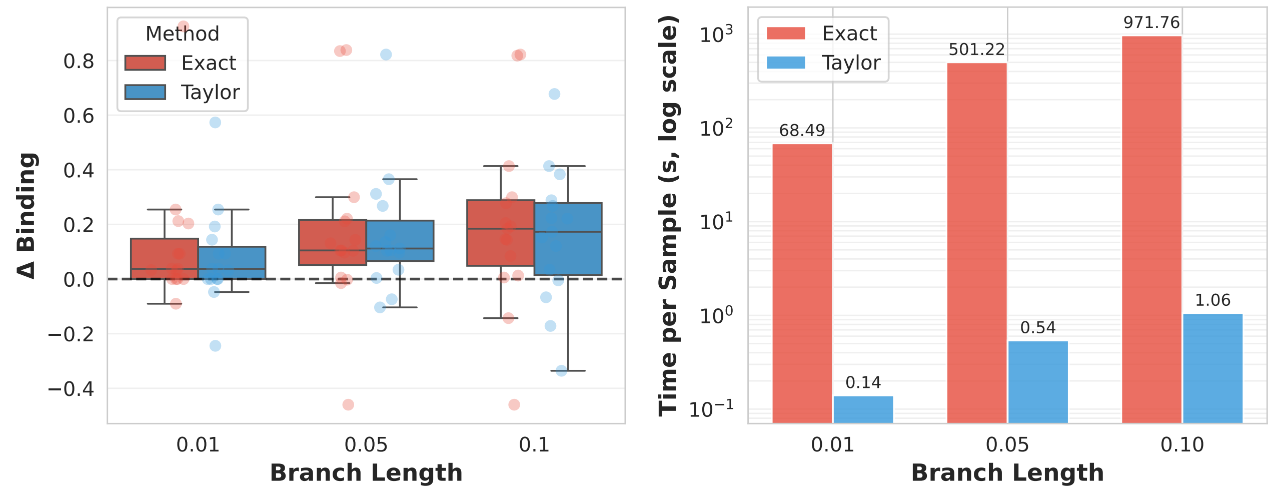

Figure S7. **TAG guidance matches exact guidance performance with 500–900× speedup**. (Left) Fitness improvements and (right) runtime comparison across branch lengths. No significant fitness differences ($p > 0.05$), but TAG is orders of magnitude faster.

Table S4. **Diversity, naturalness, and binding affinity metrics for constrained CDR optimization of SARS-CoV-1 binder.**

Method	Unique ↑	E_{dist}	IntDiv ↑	OASis ↑	Δ Bind$_{\text{mean}}$ ↑	Δ Bind$_{\text{max}}$ ↑	N_{oracle} ↓
Greedy*	0.996	5.00	6.21	0.807	0.385	0.466	2756
Genetic Algo.	0.991	4.47	7.30	0.789	0.196	0.363	4.81
AbLang-1-*PoE*	0.999	4.97	7.71	**0.849**	0.167	0.390	0.580
ESM2-*PoE*	**1.00**	4.98	**8.47**	0.802	0.149	0.355	0.580
CoSiNE (ours)	0.988	4.56	6.83	0.822	**0.198**	**0.395**	5.00

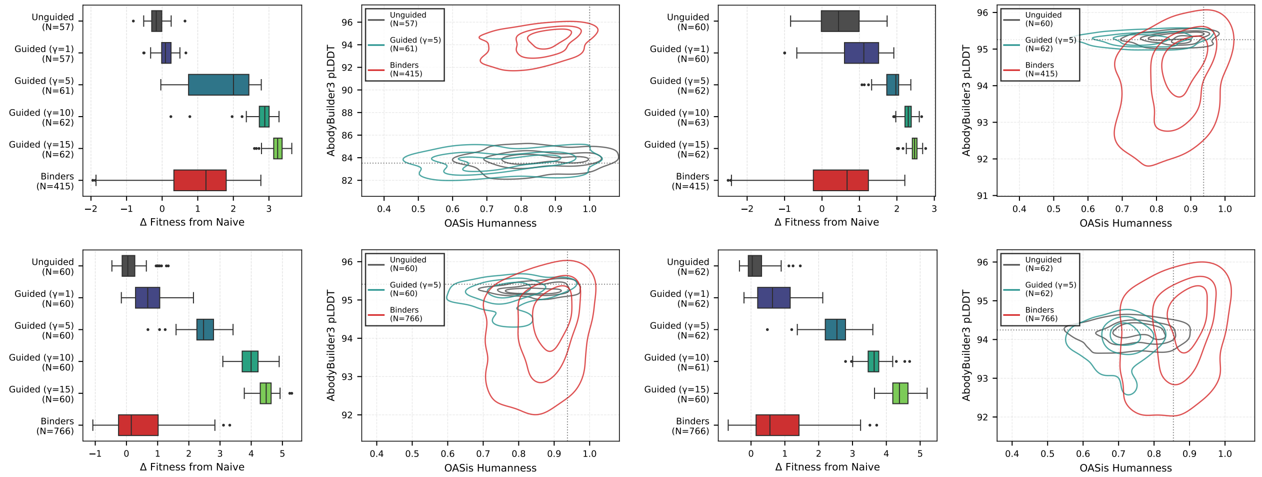

Figure S8. **Guided affinity maturation results for additional naive antibody sequences using CoV-1 (top) and CoV-2 (bottom) oracles.** We randomly selected naive IgM heavy sequences from the OAS database and recursively sampled down the tree in Figure S1.

D. Theoretical Results

D.1. First-Order Approximation of Sequential Point Mutation Process

Proposition D.1. ***(Proof of Proposition 4.1)***

Assume the per-site rate matrices $Q_\theta(x)_\ell$ are parameterized such that

$$(Q_\theta(x)_\ell)_{x_\ell, y_\ell} = \mathbf{Q}_{x,y}$$

for all x, y such that Hamming distance $\mathrm{d}(x, y) = 1$ and ℓ is the unique site where x and y differ. Then, the error between the transition probability vectors is bounded such that

$$\|P(\cdot \mid x, t) - p_\theta(\cdot \mid x, t)\|_1 \leq (\lambda t)^2 = O(t^2) \tag{10}$$

where λ is the maximum exit rate of $\mathbf{Q}$.

Proof. Notice that we can reframe the transition probabilities from the per-site rate matrices using Kronecker products as follows

$$p_\theta(\cdot \mid x, t) = \left(e^{tQ_\theta(x)_1} \otimes \cdots \otimes e^{tQ_\theta(x)_L}\right)_{x,\cdot}$$

Using the identity $e^{tA} \otimes e^{tB} = e^{t(A \oplus B)}$ we can define a **Kronecker-sum generator matrix**

$$\mathbf{Q}_\theta(x) := Q_\theta(x)_1 \oplus \cdots \oplus Q_\theta(x)_L$$

This makes a comparison between the full state space transition probabilities and per-site factorized transition probabilities more convenient

$$P(\cdot \mid x, t) = \left(e^{t\mathbf{Q}}\right)_{x,\cdot} \tag{11}$$

$$p_\theta(\cdot \mid x, t) = \left(e^{t\mathbf{Q}_\theta(x)}\right)_{x,\cdot} \tag{12}$$

Now, we can uniformize both transition kernels by picking $\lambda = \max_z\{-\mathbf{Q}_{z,z}\}$ (which is also equal to $\max_z\{-\mathbf{Q}_\theta(x)_{z,z}\}$) and defining embedded DTMCs

$$R = I + \mathbf{Q}/\lambda \tag{13}$$

$$S = I + \mathbf{Q}_\theta(x)/\lambda \tag{14}$$

Uniformization tells us

$$e^{t\mathbf{Q}} = \sum_{n=0}^{\infty} e^{-\lambda t} \frac{(\lambda t)^n}{n!} R^n, \quad e^{t\mathbf{Q}_\theta(x)} = \sum_{n=0}^{\infty} e^{-\lambda t} \frac{(\lambda t)^n}{n!} S^n,$$

Using the matching assumption for single-site mutants stated in the theorem, we know that

$$\mathbf{Q}_{x,\cdot} = \mathbf{Q}_\theta(x)_{x,\cdot} \implies R_{x,\cdot} = S_{x,\cdot}$$

Therefore, the $n = 0$ and $n = 1$ terms in the uniformization series match exactly for row x, meaning the differences start only at $n \geq 2$

$$\left(e^{t\mathbf{Q}} - e^{t\mathbf{Q}_\theta(x)}\right)_{x,\cdot} = \sum_{n=2}^{\infty} e^{-\lambda t} \frac{(\lambda t)^n}{n!} \left(R^n - S^n\right)_{x,\cdot}$$

Because $(R^n)_{x,\cdot}$ and $(S^n)_{x,\cdot}$ are valid probability distributions, $\| \left(R^n - S^n\right)_{x,\cdot} \|_1 \leq 2$, allowing us to bound the L_1 error between the true transition probability vector and per-site factorized probability vector as

$$\|P(\cdot \mid x, t) - p_\theta(\cdot \mid x, t)\|_1 = \left\| \left(e^{t\mathbf{Q}} - e^{t\mathbf{Q}_\theta(x)}\right)_{x,\cdot} \right\|_1 \leq 2 \sum_{n=2}^{\infty} e^{-\lambda t} \frac{(\lambda t)^n}{n!} = 2\left(1 - e^{-\lambda t}(1 + \lambda t)\right)$$

Recognizing that the series $1 - e^{-u}(1+u) = u^2/2 - u^3/6 + \cdots \leq u^2/2$ for $u \geq 0$ allows us to simplify the bound to[*]

$$\|P(\cdot \mid x, t) - p_\theta(\cdot \mid x, t)\|_1 \leq (\lambda t)^2 = O(t^2)$$

□

D.2. Exactness of Gillespie Sampling

Lemma D.2. ***(Proof of Lemma 4.2)*** *Let $x_0, \ldots, x_{t_{N-1}}, x_{t_N}$ be the trajectory of sequences sampled from the Gillespie procedure in Algorithm S1, using branch length t and starting sequence x_0. For all $x \in \{x_0, \ldots, x_{N-1}\}$, assuming that*

$$(Q_\theta(x)_\ell)_{x_\ell, y_\ell} = \mathbf{Q}_{x,y}$$

holds for all sequences y with Hamming distance $d(x, y) = 1$, then $x_{t_N} \sim P(\cdot \mid x_0, t)$

Proof. The proof follows from the fact that a continuous-time Markov chain is uniquely characterized by its holding time distributions and jump chain probabilities. By construction, the algorithm computes the total exit rate $\lambda_x \leftarrow -\sum_{\ell=1}^{L} Q_\theta(x)_{x_\ell, x_\ell}$ to sample the holding time. Under the lemma's condition that $(Q_\theta(x)_\ell)_{x_\ell, y_\ell} = \mathbf{Q}_{x,y}$ for all single residue mutants y, this sum is exactly equal to the target exit rate $-\mathbf{Q}_{x,x}$. Subsequently, the algorithm selects the next state y, corresponding to mutation (ℓ^*, a^*), with probability $P(\ell, a) = (Q_\theta(x)_\ell)_{x_\ell, a}/\lambda_x$. Due to the previous set of assumptions, this is identical to the transition probability $\mathbf{Q}_{x,y}/-\mathbf{Q}_{x,x}$ of the target process. Since both the exponential holding times and discrete transition probabilities match those defined by the generator $\mathbf{Q}$ at every step, the simulated trajectory is a statistically exact realization of the true process, ensuring x_{t_N} is distributed according to $P(\cdot \mid x_0, t)$. □

D.3. Relation Between Fixation Probability and Relative Fitness

Following Kimura (1962), we define P_{fix} for a haploid population (which we use to describe affinity maturation due to asexual reproduction of B cells and allelic exclusion, which ensures that only one allele is expressed per B cell) as

$$P_{\text{fix}}(x \to y) = \frac{1 - e^{-2s_{xy}}}{1 - e^{-2N_e s_{xy}}}, \tag{15}$$

where x is the wildtype allele, y is a newly introduced allele, $s_{xy} = F_y - F_x$ is the selective advantage of allele y over allele x, and N_e is the effective population size. Crucially, the fixation probability expression is monotonic with respect to s_{xy}, which explains why the selection score calculated by Equation (5) shows strong Spearman correlation with empirical relative fitness measurements.

[*]The constant λ corresponds to the maximum total substitution rate out of any sequence state. Under standard sequential point-mutation models, this rate scales at most linearly with sequence length and is bounded by the sum of per-site mutation rates.
